# Exploratory Comparison of Routine Inflammatory Biomarkers in Nasopharyngeal Carcinoma and Benign Adenoid-Associated Pathology

**DOI:** 10.3390/diseases14100365

**Published:** 2026-10-02

**Authors:** Darius Radu Roman, Carmen Delia Nistor Cseppento, Dana Carmen Zaha, Timea Claudia Ghitea, Alexia Manole, Dana Zdremtan, Alexandru Chioreanu, Felicia Manole

**Affiliations:** 1 Doctoral School of Biomedical Sciences, Faculty of Medicine and Pharmacy, University of Oradea, 410087 Oradea, Romania; roman.dariusradu@student.uoradea.ro; 2 Department of Psycho-Neuroscience and Recovery, Faculty of Medicine and Pharmacy, University of Oradea, 410073 Oradea, Romania; dcseppento@uoradea.ro; 3 Department of Preclinical Disciplines, Faculty of Medicine and Pharmacy, University of Oradea, 410073 Oradea, Romania; danaczaha@gmail.com; 4 Department of Pharmacy, Faculty of Medicine and Pharmacy, University of Oradea, 410073 Oradea, Romania; 5 Faculty of Medicine and Pharmacy, University of Oradea, 410068 Oradea, Romania; manole.alexia@student.uoradea.ro; 6 Department of Biochemistry, “Vasile Goldis” Western University, B-dul Revolutiei Nr. 96, 310025 Arad, Romania; zdremtan.dana@uvvg.ro (D.Z.); chioreanu.alexandru@uvvg.ro (A.C.); 7 Medicine Department, Faculty of Medicine and Pharmacy, University of Oradea, 410068 Oradea, Romania; fmanole@uoradea.ro

**Keywords:** nasopharyngeal carcinoma, C-reactive protein, erythrocyte sedimentation rate, leukocyte count, systemic inflammation, endoscopy, adenoid-associated pathology, retrospective study

## Abstract

Background: Nasopharyngeal carcinoma (NPC) may be accompanied by systemic inflammatory changes, but routine inflammatory markers are nonspecific and have not been validated as diagnostic tests for NPC. Methods: This exploratory retrospective comparison screened 178 adults and included 72 eligible records (36 histopathologically confirmed NPC and 36 histologically confirmed benign adenoid-associated pathology); 106 records were excluded because of incomplete endoscopic or laboratory records (*n* = 41), hematological malignancy (*n* = 15), severe hepatic failure (*n* = 11), or a documented systemic inflammatory condition unrelated to the nasopharyngeal presentation (*n* = 39). CRP, erythrocyte sedimentation rate (ESR), leukocyte count, selected biochemical variables, and an investigator-derived loco-regional clinical/endoscopic severity score were compared. Prespecified sensitivity analyses included age- and sex-adjusted rank regression, restriction to the overlapping age range (39–52 years), group-stratified correlations, and partial rank correlations adjusted for diagnostic group, age, and sex. Results: The NPC group was younger than the benign group (38.1 ± 7.6 vs. 56.1 ± 8.3 years; *p* < 0.001). CRP, ESR, leukocyte count, uric acid, and the loco-regional score were higher in the NPC group (all *p* < 0.01). The principal differences persisted in the overlapping-age subset (NPC *n* = 17; benign *n* = 16; all *p* < 0.001) and in age- and sex-adjusted rank analyses. Pooled correlations were attenuated or changed after stratification and covariate adjustment, indicating that part of the pooled association reflected between-group separation. Exploratory ROC AUCs were 1.000 for CRP, leukocyte count, and the loco-regional score and 0.948 for ESR; these values were derived and evaluated in the same selected cohort. In separate Firth models adjusted for age and sex, CRP, leukocyte count, and the loco-regional score remained associated with NPC, whereas ESR did not (*p* = 0.200). Conclusions: The study identifies marked between-group differences in this selected retrospective cohort but does not establish diagnostic biomarkers or clinical cut-offs. Independent prospective evaluation, standardized sampling, and fuller control of inflammatory and tumor-related confounding are required.

## 1. Introduction

Nasopharyngeal carcinoma (NPC) is an epithelial malignancy with distinctive epidemiology, anatomical behavior, and etiological associations. Although uncommon globally, it remains clinically important because early symptoms may be nonspecific and the disease may present with locally advanced or nodal involvement [1,2].

NPC pathogenesis is multifactorial and involves host susceptibility, environmental exposures, and Epstein–Barr virus (EBV), particularly in endemic populations [3,4,5]. Systemic inflammatory markers may be elevated in cancer, but they are nonspecific and can also reflect infection, medication exposure, tissue injury, smoking, alcohol use, comorbid disease, or tumor burden.

The nasopharynx contains abundant lymphoid tissue and is exposed continuously to inhaled antigens and microorganisms. Adult adenoid enlargement and chronic adenoid-associated inflammation may therefore produce symptoms, inflammatory findings, and endoscopic appearances that overlap with malignant disease [6,7,8].

Among the laboratory parameters most frequently evaluated in clinical practice, C-reactive protein (CRP), erythrocyte sedimentation rate (ESR), and leukocyte count are inexpensive and widely available markers of systemic inflammation. Several studies have reported associations between elevated inflammatory markers and adverse outcomes in patients with NPC, although their diagnostic role remains uncertain [9,10]. C-reactive protein (CRP), erythrocyte sedimentation rate (ESR), and leukocyte count are widely available measures of systemic inflammation. Published NPC studies have primarily evaluated their prognostic rather than diagnostic value, and elevated CRP has been associated with disease burden and adverse outcomes [11,12,13,14]. These observations do not establish specificity for NPC.

Nasopharyngeal endoscopy is central to lesion assessment and biopsy targeting, but benign and malignant abnormalities may overlap, and some early cancers may be occult on endoscopy. Histopathology remains the reference standard, with imaging providing complementary information [15,16,17].

This study aimed to compare routinely measured inflammatory, hematological, biochemical, and loco-regional clinical/endoscopic variables between histopathologically confirmed NPC and histologically confirmed benign adenoid-associated pathology. Secondary objectives were to examine whether the main comparisons persisted after accounting for age and sex, to distinguish pooled from within-group correlations, and to describe the exploratory discrimination observed in this dataset without presenting it as formally established diagnostic performance.

## 2. Materials and Methods

### 2.1. Study Design and Patient Selection

This retrospective observational comparison assessed adult patients evaluated for suspected nasopharyngeal pathology between January 2022 and December 2025. Group allocation was based exclusively on histopathology. Screening, eligibility, and exclusion data were reconstructed from the source study records.

A total of 178 adults evaluated for suspected nasopharyngeal pathology were screened for eligibility. Of these, 41 were excluded because endoscopic or laboratory records were incomplete, 15 because of hematological malignancy, 11 because of severe hepatic failure, and 39 because of a documented systemic inflammatory condition unrelated to the nasopharyngeal presentation. The remaining 72 records comprised 36 histopathologically confirmed NPC cases and 36 histologically confirmed benign adenoid-associated cases. No age or sex matching was used. Consequently, the benign group is used for descriptive context rather than as a population-based control group.

The analytic inclusion criteria were adulthood, histopathological confirmation, and availability of demographic, laboratory, endoscopic/clinical score, and pathology data. The manuscript cannot verify additional exclusion criteria against the supplied dataset because active infection, systemic inflammatory disease, medication exposure, recent procedures, comorbidities, EBV status, and tumor stage were not recorded as analyzable fields.

No a priori sample-size calculation was available. All analyses were exploratory and hypothesis-generating. Reporting was revised with reference to the STROBE recommendations for observational studies [12], while explicitly identifying items that could not be reconstructed from the de-identified dataset.

### 2.2. Baseline Characteristics

Age and sex were available for all 72 participants. The dataset also contained coded smoking and alcohol variables, with identical numeric distributions in the two groups; however, a data dictionary defining the codes, exposure intensity, duration, and timing was not supplied. These variables were therefore not interpreted as verified clinical exposures.

Recent infection or fever, antibiotics, corticosteroids, non-steroidal anti-inflammatory drugs, medication use, comorbidities, EBV DNA/EBER/serology, nutritional status, and NPC stage were absent from the analytic dataset. These factors could not be incorporated into adjusted or exclusion-based sensitivity analyses and are treated as potential sources of residual confounding.

### 2.3. Clinical and Endoscopic Assessment

All participants underwent flexible nasopharyngeal endoscopy and routine otorhinolaryngological assessment. Documented features used for the exploratory score included mucosal edema, irregular mucosal lesion, focal necrosis, tubal obstruction, extensive infiltrative lesion, and laterocervical adenopathy. Because laterocervical adenopathy is a clinical/imaging rather than endoscopic finding, the measure is termed a loco-regional clinical/endoscopic severity score.

All flexible nasopharyngeal endoscopies were performed by experienced otorhinolaryngologists, who also interpreted the endoscopic findings. The ENT team involved in the examinations is fully represented in the author list. No separate reviewer was involved, and no formal assessment procedure was performed. A formal blinded-rating procedure was not documented and cannot be claimed. Duplicate independent ratings were unavailable; therefore, interobserver agreement was not calculated.

### 2.4. Loco-Regional Clinical/Endoscopic Severity Score

The investigator-derived score assigned 1 point for mucosal edema, 2 for an irregular mucosal lesion, 3 for focal necrosis, 4 for partial or complete tubal obstruction, 5 for an extensive infiltrative lesion, and 1 for clinically or radiologically documented laterocervical adenopathy. The weights were intended to represent an ordinal increase in presumed loco-regional severity; they were not estimated from the present data and were not empirically tested. Irregular mucosal lesion and extensive infiltrative lesion were treated as mutually exclusive, producing a theoretical range of 0–11. The score is used only as a descriptive exploratory measure and not as a diagnostic instrument.

### 2.5. Laboratory and Inflammatory Assessment

Peripheral venous blood was reported as collected at admission before oncological treatment or major surgery. The supplied dataset did not contain collection dates or times relative to endoscopy, biopsy, fever, or other procedures.

Variables analyzed were CRP, ESR, total leukocyte count, platelet count, gamma-glutamyl transferase (GGT), aspartate aminotransferase (AST), alanine aminotransferase (ALT), and uric acid. All measurements were performed in the hospital laboratory using the same institutional equipment and standard operating procedures throughout the study period. Routine biochemical determinations were performed with commercially available reagent kits, predominantly supplied by Merck, in accordance with the manufacturer’s instructions. Calibration and internal quality-control procedures followed the laboratory’s standard operating procedures and the relevant manufacturer’s recommendations. Differential leukocyte labels and derived NLR/PLR/SII values were excluded because their identity and calculation could not be verified. One total leukocyte count was recorded as zero in the analytical dataset. This entry was treated as missing rather than interpreted as a measured leukocyte count, leaving 71 observations for leukocyte-specific regression, ROC, and correlation analyses (*n* = 71).

### 2.6. Statistical Analysis

Continuous variables were assessed graphically and with the Shapiro–Wilk test. Normally distributed variables are presented as mean ± standard deviation, and skewed variables as median (interquartile range). Between-group comparisons were performed using independent-samples *t*-tests or Mann–Whitney U tests as appropriate; categorical variables were assessed using Fisher’s exact test.

No multiplicity correction was applied because the analyses were exploratory. All *p*-values are two-sided and should be interpreted as descriptive evidence rather than confirmatory hypothesis tests.

Spearman correlations were calculated in the pooled cohort and separately within the NPC and benign groups. To assess the influence of between-group separation and age, partial rank correlations were obtained by correlating rank residuals after adjustment for diagnostic group, age, and sex.

Separate Firth penalized logistic regression models evaluated each biomarker in relation to histopathologically confirmed NPC after adjustment for age and sex. Because each model contained only one biomarker plus age and sex, results indicate association after adjustment for age and sex; they do not demonstrate independence from the other inflammatory variables.

Exploratory ROC analyses were performed for CRP, ESR, leukocyte count, and the loco-regional clinical/endoscopic score. AUCs, 95% confidence intervals, Youden-derived cut-offs, sensitivity, and specificity were calculated in the same cohort. These estimates were not independently tested and were not intended to define clinical thresholds.

Sensitivity analyses included (i) rank-based linear regression of each principal marker on diagnostic group, age, and sex with HC3 robust standard errors and (ii) Mann–Whitney comparisons restricted to the common age range of 39–52 years. Primary values and Firth estimates were independently reproduced from the supplied CSV using Python 3.11 (pandas 3.0.6., SciPy 1.18.1., statsmodels 0.15.0., and scikit-learn 1.9.1.); the manuscript’s original software record should be retained by the authors if different.

### 2.7. Ethical Considerations

The study was conducted in accordance with the ethical principles of the Declaration of Helsinki and was approved by the Institutional Ethics Committee of the University of Oradea and the affiliated clinical institution (Approval No. 80/31 March 2026).

Because of the retrospective observational design and the use of anonymized routinely collected clinical data, the requirement for informed consent was waived in accordance with institutional regulations.

## 3. Results

### 3.1. Clinical and Laboratory Characteristics of the Study Groups

Of the 178 adults screened, 106 were excluded: 41 because endoscopic or laboratory records were incomplete, 15 because of hematological malignancy, 11 because of severe hepatic failure, and 39 because of a documented systemic inflammatory condition unrelated to the nasopharyngeal presentation. The final cohort included 72 adults: 36 with histopathologically confirmed NPC and 36 with histologically confirmed benign adenoid-associated pathology.

The NPC group was younger than the benign group (38.1 ± 7.6 vs. 56.1 ± 8.3 years; *p* < 0.001). Sex distribution was similar (32/36 vs. 31/36 coded male; Fisher’s exact *p* = 1.000). The numeric smoking and alcohol codes had identical group distributions, but their clinical definitions were unavailable and were not interpreted.

All participants underwent flexible nasopharyngeal endoscopy and histopathological assessment. Histopathology alone defined the groups; endoscopic and laboratory variables were not used to establish the reference diagnosis.

CRP, ESR, leukocyte count, uric acid, and the loco-regional clinical/endoscopic score were higher in the NPC group. Platelet count, GGT, AST, and ALT did not differ significantly. Differential leukocyte variables and derived inflammatory indices were not reported because the supplied labels and calculations could not be verified.

The loco-regional clinical/endoscopic score was 7.25 ± 2.31 in the NPC group and 2.08 ± 1.05 in the benign group; medians were 6.5 (5.75–8.0) and 2.0 (1.0–3.0), respectively (*p* < 0.001). This investigator-derived measure is descriptive and includes cervical adenopathy in addition to endoscopic findings.

The complete age- and sex-adjusted Firth regression results are reported in Table 1.

Pathological confirmation and endoscopic assessment were available for all included patients. The terminology “benign adenoid-associated pathology” is used consistently for the comparison group because more specific labels such as adenoid hypertrophy or chronic adenoiditis were not uniformly documented in the supplied analytic dataset.

In age- and sex-adjusted rank analyses, the diagnostic group remained associated with CRP, ESR, leukocyte count, and the loco-regional score (all *p* < 0.001). Restriction to the overlapping age range of 39–52 years included 17 NPC and 16 benign cases; group differences persisted for CRP (*p* < 0.001), ESR (*p* < 0.001), leukocyte count (*p* < 0.001), and the score (*p* < 0.001). These analyses reduce but do not eliminate confounding or selection bias (Table 2).

**Figure 1:**
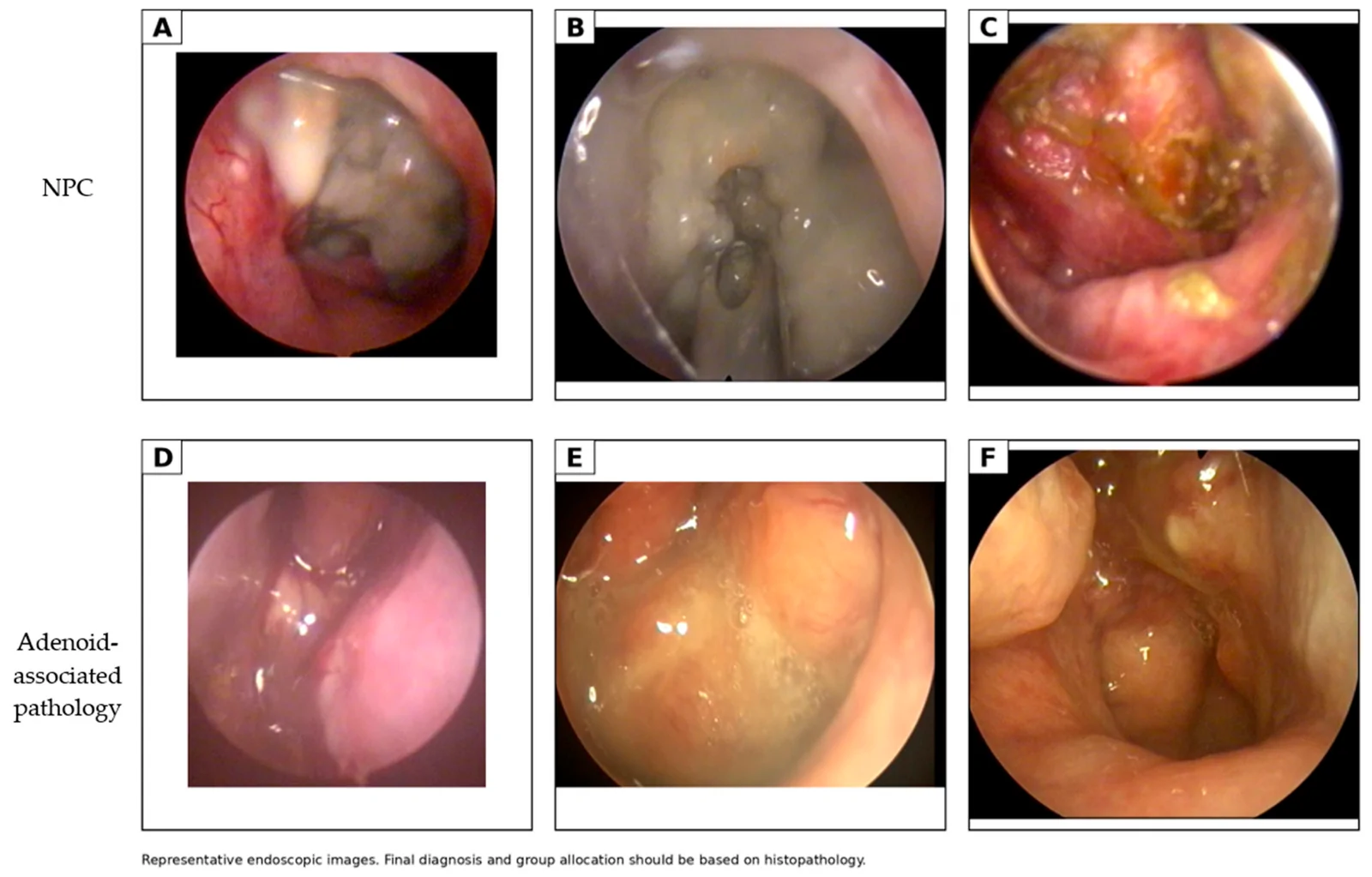
Representative nasopharyngeal endoscopic findings in the study groups. (**A**–**C**) Endoscopic appearances in patients with histopathologically confirmed nasopharyngeal carcinoma (NPC), shown in the upper row. (**D**–**F**) Endoscopic appearances in patients with histopathologically confirmed benign adenoid-associated pathology, shown in the lower row. Panels are arranged from left to right within each row. Images are illustrative only; the final diagnosis was based on histopathology. Clinical and endoscopic assessments were performed and interpreted by experienced otorhinolaryngologists who are co-authors and members of the ENT team. No formal blinded duplicate rating or independent assessment by separate reviewers was performed.

In separate age- and sex-adjusted Firth models, CRP, leukocyte count, and the loco-regional clinical/endoscopic score remained associated with NPC; ESR did not (*p* = 0.200). These are not mutually adjusted biomarker models (Figure 1).

Table 3 summarizes directly measured laboratory variables and the exploratory loco-regional clinical/endoscopic score. One total leukocyte entry recorded as zero was treated as missing and excluded from leukocyte-specific analyses. Consequently, the leukocyte summary for the benign group included 35 observations (n = 35), and the leukocyte regression model included 71 observations across both diagnostic groups (n = 71).

**Figure 2:**
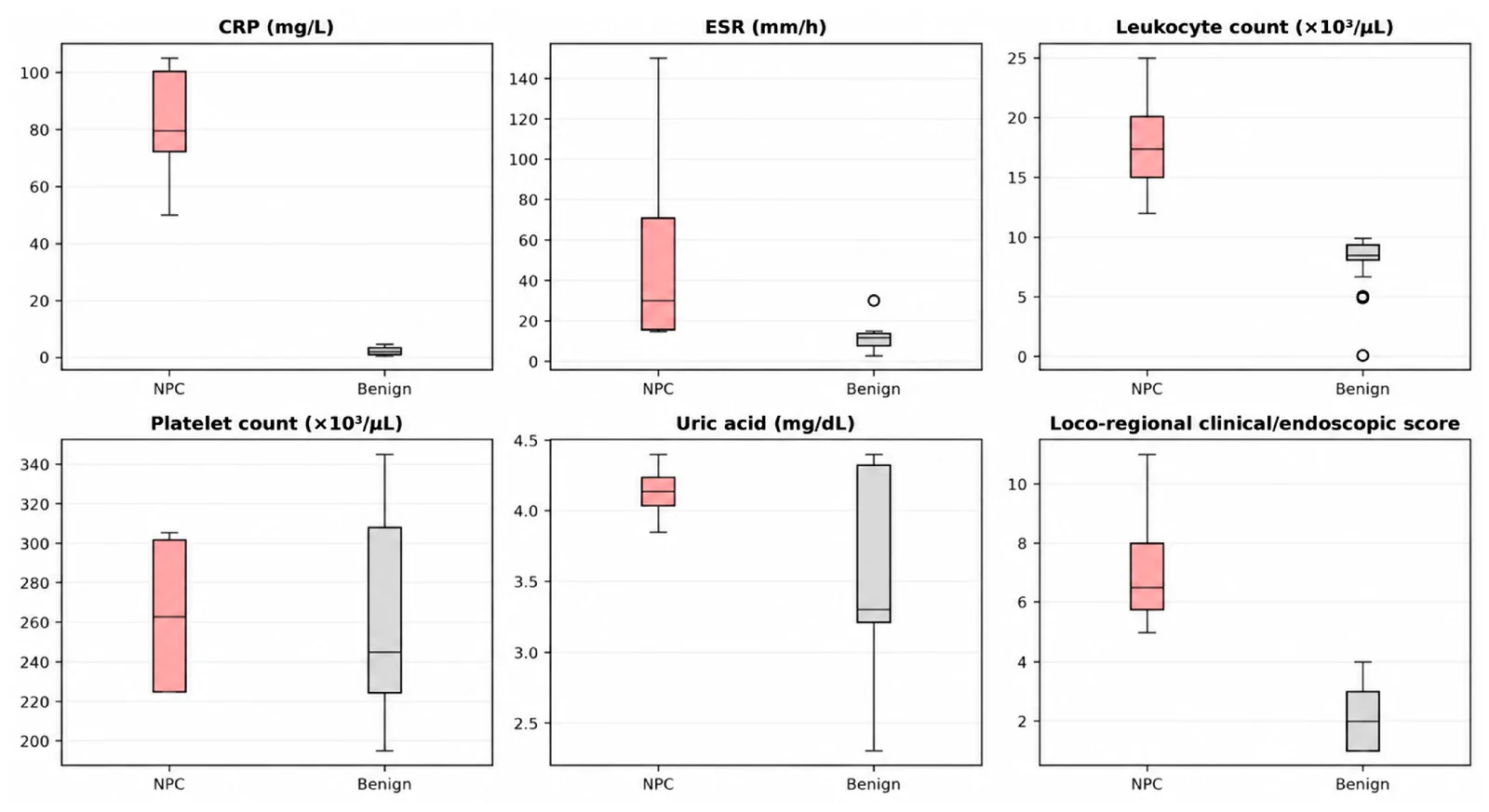
Distribution of CRP, ESR, leukocyte count, platelet count, uric acid, and the exploratory loco-regional clinical/endoscopic score in patients with nasopharyngeal carcinoma (NPC) and benign adenoid-associated pathology. Horizontal lines within boxes indicate medians; boxes represent the interquartile range (IQR), and whiskers extend to the most extreme observations within 1.5 × IQR of the box boundaries. Hollow circles indicate observations beyond these limits (outliers). One leukocyte entry recorded as zero was treated as missing and excluded from the leukocyte plot and corresponding analyses (*n* = 71); all other panels include 72 observations. Between-group comparisons used Mann–Whitney U tests. CRP, C-reactive protein; ESR, erythrocyte sedimentation rate.

CRP, leukocyte count, and the loco-regional score remained associated with NPC after adjustment for age and sex; ESR did not. No model established independence relative to the other biomarkers (Figure 2).

### 3.2. Correlation Analysis of Laboratory and Endoscopic Variables

Spearman correlations among CRP, ESR, leukocyte count, and the loco-regional score were calculated in the pooled cohort and separately by diagnostic group. The leukocyte entry recorded as zero was treated as missing and excluded from all correlations involving leukocyte count.

Pooled correlations were strong (ρ = 0.785–0.870; all *p* < 0.001). Within the NPC group, all six principal pairwise correlations remained positive and significant (ρ = 0.678–0.950; all *p* < 0.001). Within the benign group, five of six correlations were not significant; the exception was an inverse correlation between leukocyte count and the loco-regional score (ρ = −0.837, *p* < 0.001).

After adjustment for diagnostic group, age, and sex, CRP remained correlated with ESR (partial ρ = 0.347, *p* = 0.003) and leukocyte count (partial ρ = 0.432, *p* < 0.001), and ESR remained correlated with leukocyte count (partial ρ = 0.289, *p* = 0.015) and the score (partial ρ = 0.585, *p* < 0.001). CRP–score (*p* = 0.056) and leukocyte–score (*p* = 0.290) associations were no longer statistically significant.

The attenuation and heterogeneity of these associations confirm that pooled correlations partly reflected separation between the two diagnostic groups. They should not be interpreted as uniform within-group biological relationships (Table 4).

**Figure 3:**
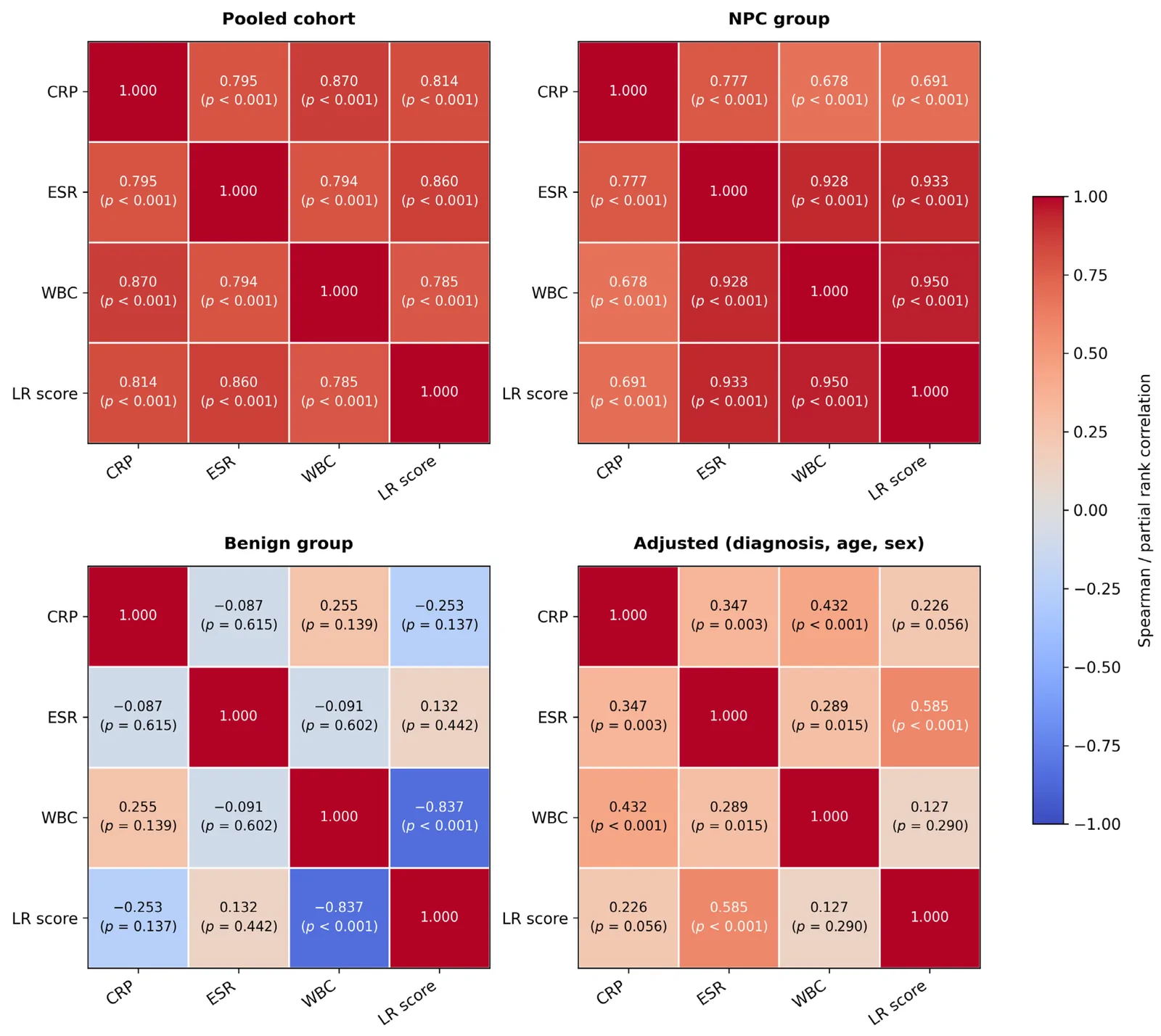
Pooled, group-specific, and adjusted rank correlations among CRP, ESR, leukocyte count, and the exploratory loco-regional clinical/endoscopic score. Panels show Spearman correlations in the pooled cohort (upper left), NPC group (upper right), and benign group (lower left), and partial rank correlations adjusted for diagnostic group, age, and sex (lower right). Cells display correlation coefficients and corresponding *p*-values in parentheses; diagonal cells indicate self-correlations. The color scale indicates the direction and magnitude of correlations, from negative (blue) to positive (red). Analyses involving leukocyte count excluded the entry recorded as zero. CRP, C-reactive protein; ESR, erythrocyte sedimentation rate; WBC, total leukocyte count; LR score, exploratory loco-regional clinical/endoscopic score; NPC, nasopharyngeal carcinoma.

Figure 3 and Table 4 show that the pooled pattern was not reproduced uniformly within each diagnostic group.

### 3.3. Exploratory Diagnostic Performance of Routinely Measured Biomarkers

ROC analysis was used only to describe separation between the two histopathologically defined groups in the present derivation dataset. It was not designed to evaluate replacement of biopsy or performance in an unselected diagnostic population.

CRP, leukocyte count, and the loco-regional clinical/endoscopic score each produced AUC = 1.000; ESR produced AUC = 0.948 (95% CI 0.893–0.990). The leukocyte entry recorded as zero was treated as missing and excluded from the leukocyte ROC analysis, leaving 71 observations (n = 71).

AUCs of 1.000 are unusually high and likely reflect the selected, balanced, small cohort and marked between-group separation. Cut-offs, sensitivity, and specificity were optimized and evaluated in the same dataset without resampling or testing in an independent cohort; they are therefore exploratory and may be substantially optimistic (Table 5).

**Table 5:** Exploratory receiver operating characteristic analysis in the derivation cohort.

Marker	AUC	95% CI	Optimal Cut-Off	Sensitivity	Specificity	*p*-Value
CRP (mg/L)	1.000	1.000–1.000	50.0	1.000	1.000	<0.001
ESR (mm/h)	0.948	0.893–0.990	15.0	1.000	0.833	<0.001
Leukocyte count (×10^3^/µL), *n* = 71	1.000	1.000–1.000	12.03	1.000	1.000	<0.001
Loco-regional clinical/endoscopic score	1.000	1.000–1.000	5	1.000	1.000	<0.001

NPC was the positive outcome. Youden cut-offs were derived and evaluated in the same cohort. CRP, ESR, and score analyses used *n* = 72; leukocyte analysis used *n* = 71. These results do not constitute established diagnostic thresholds.

**Figure 4:**
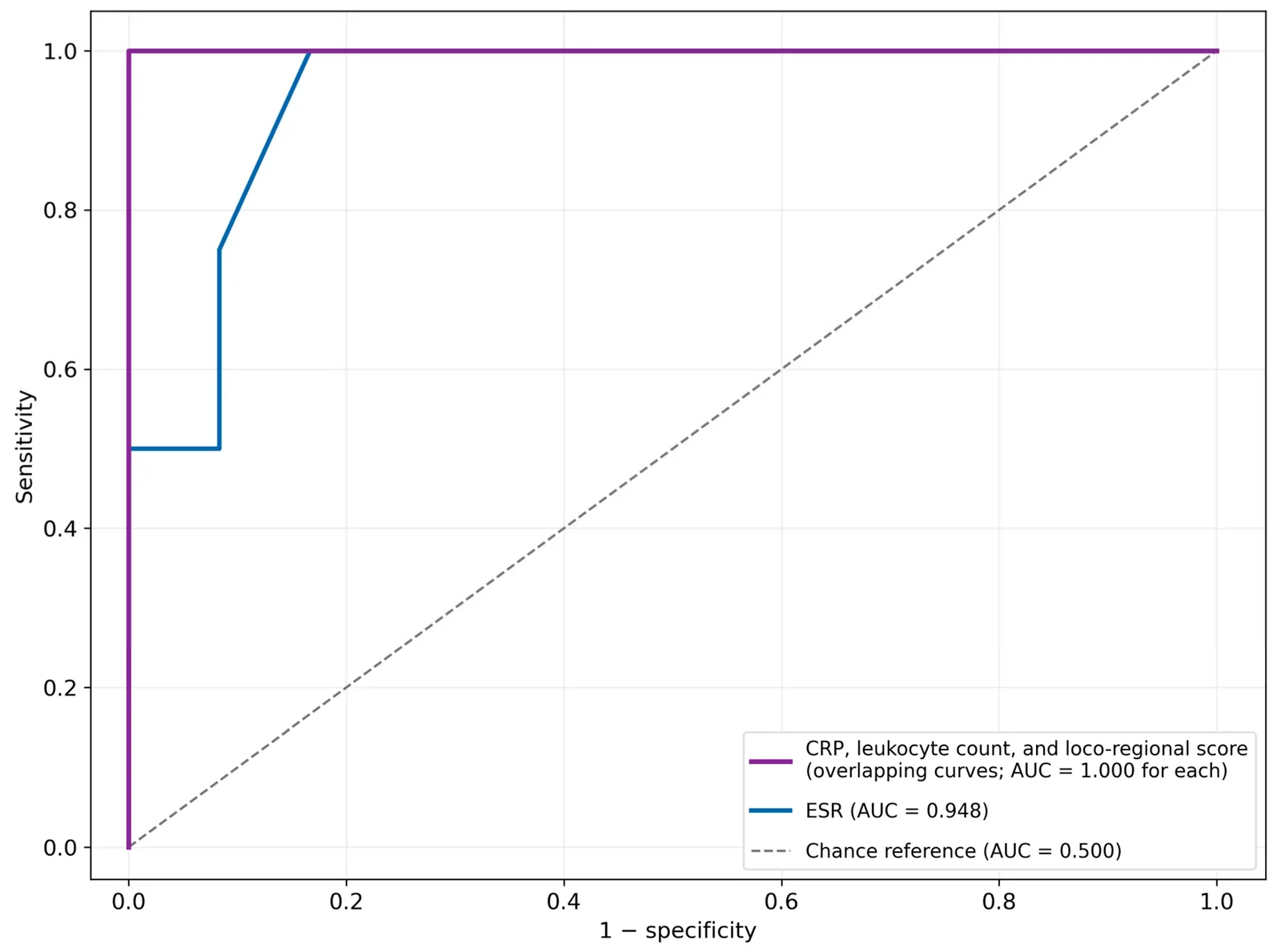
Exploratory receiver operating characteristic (ROC) curves for CRP, ESR, leukocyte count, and the loco-regional clinical/endoscopic score in the selected derivation cohort. The curves for CRP, leukocyte count, and the loco-regional score overlap completely (purple line; AUC = 1.000 for each). The blue curve represents ESR (AUC = 0.948). The dashed gray diagonal represents chance-level discrimination (AUC = 0.500). The leukocyte entry recorded as zero was excluded from the leukocyte analysis. Complete separation in this selected derivation cohort should not be interpreted as established diagnostic accuracy or as evidence that these variables can replace histopathology. CRP, C-reactive protein; ESR, erythrocyte sedimentation rate; AUC, area under the ROC curve.

The exploratory ROC curves are shown in Figure 4; independent prospective evaluation is required before any clinical interpretation.

### 3.4. Exploratory Age- and Sex-Adjusted Logistic Regression Analysis

Separate Firth penalized logistic regression models were fitted for each predictor and adjusted for age and sex. Penalization was used because several predictors showed complete or near-complete separation.

CRP (OR 3.45 per 10 mg/L, *p* = 0.003), leukocyte count (OR 4.66 per 1 × 10^3^/µL, *p* = 0.014), and the loco-regional score (OR 18.07 per point, *p* = 0.010) remained associated with NPC after age and sex adjustment. ESR was not significant (OR 1.31 per 10 mm/h, *p* = 0.200). The leukocyte model used *n* = 71; all other models used *n* = 72.

Because the biomarkers were modeled separately, the results do not establish that CRP or leukocyte count is independently associated with NPC relative to ESR or the other inflammatory variables (Table 6).

## 4. Discussion

This exploratory retrospective comparison found higher CRP, ESR, leukocyte count, uric acid, and loco-regional clinical/endoscopic scores in the NPC group. The principal differences persisted after age and sex adjustment and within the overlapping age range, but the selected 36/36 sampling structure, incomplete clinical characterization, and lack of an independent evaluation cohort limit interpretation.

The CRP, ESR, and leukocyte findings are compatible with a greater systemic inflammatory burden in the NPC group. Prior NPC literature supports a prognostic association for elevated CRP but does not establish that routine inflammatory markers are specific diagnostic biomarkers [14,17,18]. In the present dataset, the extreme CRP and leukocyte separation is unusual and may reflect disease severity, selection, infection, medication exposure, recent procedures, or other unmeasured factors.

Age was a major measured confounder because the NPC group was approximately 18 years younger on average. Rank-based age/sex adjustment and restriction to ages 39–52 years produced similar group differences, which argues against age alone explaining the results. Nevertheless, residual confounding remains likely because the groups were not matched and the overlap analysis was small.

The pooled correlation analysis initially suggested uniformly strong relationships among inflammatory markers and the loco-regional score. Stratified and partial analyses showed a more complex pattern: several correlations persisted in NPC; most were absent in the benign group, and some pooled score associations were no longer significant after adjustment. Thus, pooled coefficients partly measured diagnostic-group separation rather than a common biological relationship [19,20,21].

The investigator-derived score was higher in NPC, but its numerical weights were based on presumed ordinal severity, not empirical derivation or formal testing. Inclusion of cervical adenopathy means that it is not a purely endoscopic score. Clinical and endoscopic assessments were performed and interpreted by experienced otorhinolaryngologists who are co-authors and members of the ENT team; no separate reviewer was involved, and no formal assessment procedure was performed. A formal blinded duplicate-rating procedure and interobserver-agreement assessment were not available. The measure should therefore be considered a descriptive loco-regional index only.

Endoscopy remains important for lesion visualization and biopsy targeting, while histopathology is required for definitive diagnosis, and imaging may reveal disease not apparent endoscopically [15,16]. The present score and laboratory variables cannot replace this diagnostic pathway.

The AUCs of 1.000 for CRP, leukocyte count, and the loco-regional score describe complete separation in this dataset. Because the same small, balanced cohort was used to select cut-offs and evaluate performance, these estimates are at high risk of optimism. They should not be generalized to consecutive patients, early disease, or populations with active infection and other inflammatory conditions.

**Figure 5:**
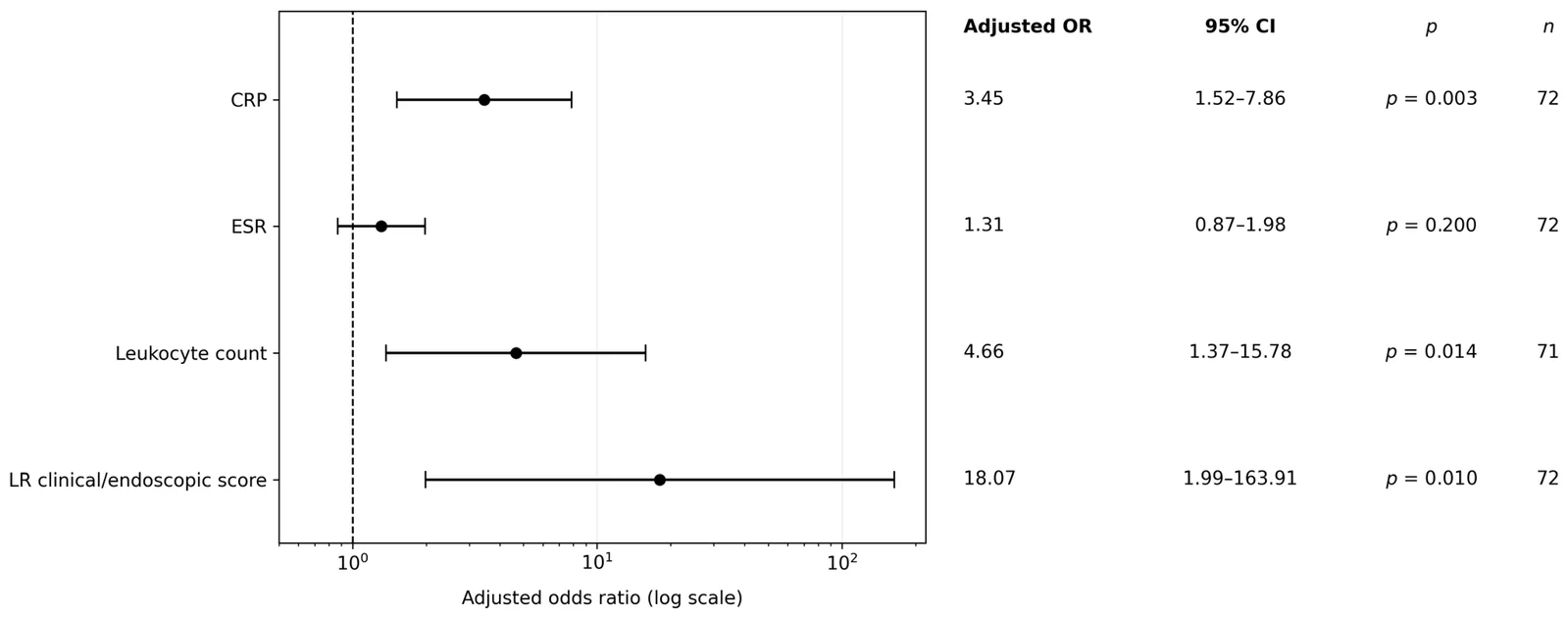
Associations with NPC in separate age- and sex-adjusted Firth penalized logistic regression models. Points represent adjusted odds ratios (ORs), and horizontal bars indicate 95% confidence intervals (CIs). The vertical dashed line marks OR = 1, indicating no association; the horizontal axis uses a logarithmic scale. CRP, leukocyte count, and the exploratory loco-regional clinical/endoscopic score were associated with NPC after adjustment for age and sex, whereas ESR was not statistically significant. The leukocyte model excluded the entry recorded as zero (*n* = 71); the other models included 72 observations. Models were exploratory and did not mutually adjust the biomarkers. CRP, C-reactive protein; ESR, erythrocyte sedimentation rate; LR, loco-regional; NPC, nasopharyngeal carcinoma.

Separate Firth penalized logistic regression models were fitted for CRP, ESR, leukocyte count, and the exploratory loco-regional clinical/endoscopic score, with each model adjusted for age and sex. The leukocyte entry recorded as zero was treated as missing, leaving 71 observations for the leukocyte model (*n* = 71); the other models included 72 observations (Table 6/Figure 5). Because each model included only one biomarker or score alongside age and sex, the estimates reflect age- and sex-adjusted associations and do not establish independence from the other biomarkers [22,23].

Data quality remains an important limitation. Differential leukocyte labels and the derived NLR, PLR, and SII calculations could not be reliably reconciled with the available dataset columns and were therefore excluded from the analyses. One total leukocyte entry recorded as zero was treated as missing and excluded from analyses involving leukocyte count. Two additional low values (0.10 and 0.12 × 10³/µL) were retained as recorded. Their validity could not be established from the analytical dataset alone and requires verification against the original laboratory reports.

Potential inflammatory confounders could not be assessed because the analytic dataset lacked recent infection or fever, antibiotic, corticosteroid and NSAID exposure, recent biopsy or other procedures, and relevant comorbidities. Smoking and alcohol were numerically coded without a supplied codebook. These omissions prevent causal or disease-specific interpretation.

EBV status, tumor stage, T/N category, metastatic status, tumor volume, and histological subtype were unavailable. Accordingly, the analysis cannot determine whether inflammatory-marker elevations were related to EBV biology, tumor burden, or stage. Future studies should collect these variables prospectively [10,24,25,26,27].

The comparison group is consistently termed “benign adenoid-associated pathology.” Adult adenoid tissue may show chronic inflammation, follicular hyperplasia, or secondary epithelial changes [5,6,7], but the more specific terms “adenoid hypertrophy”, “chronic adenoiditis”, and “lymphoid hyperplasia” should not be used interchangeably without case-level histopathological confirmation [28,29,30,31,32,33,34].

Additional limitations include the single-center retrospective design, small sample, selected case-comparison structure, lack of a population-based control group, no multiplicity correction, and the absence of formal blinded duplicate scoring and interobserver-reliability assessment. The study should be viewed as a selected exploratory case-comparison analysis rather than a diagnostic-accuracy study with established performance.

A prospective study should enroll consecutive patients; prespecify eligibility and score definitions; document observer blinding and reliability; standardize blood sampling before biopsy or treatment; record inflammatory confounders, EBV, and stage; verify laboratory variables; and independently evaluate any model or threshold in a separate cohort.

## 5. Conclusions

In this selected exploratory cohort, histopathologically confirmed NPC was associated with higher CRP, ESR, leukocyte count, uric acid, and an investigator-derived loco-regional clinical/endoscopic score than benign adenoid-associated pathology. The principal group differences persisted in age-focused sensitivity analyses, but pooled correlations were partly explained by between-group separation. The AUCs and cut-offs were derived and evaluated in the same small dataset and do not establish diagnostic biomarkers. Consecutive prospective recruitment, comprehensive confounder assessment, standardized measurement, and independent evaluation are required before clinical use.

## Figures and Tables

**Table 1:** Demographic and baseline characteristics of the study groups.

Variable	NPC Group, *n* = 36	Benign Adenoid-Associated Group, *n* = 36	*p*-Value
Age, years	38.1 ± 7.6	56.1 ± 8.3	<0.001
Male sex, *n* (%)	32 (88.9%)	31 (86.1%)	1.000
Female sex, *n* (%)	4 (11.1%)	5 (13.9%)	—
Histopathological assessment, *n* (%)	36 (100%)	36 (100%)	—
Flexible nasopharyngeal endoscopy, *n* (%)	36 (100%)	36 (100%)	—

Data are mean ± standard deviation or *n* (%). Age was compared using an independent-samples *t*-test and sex using Fisher’s exact test. Histopathology defined the groups and was not tested inferentially. Smoking and alcohol codes were not labeled in the supplied data dictionary and should not be interpreted until verified. NPC, nasopharyngeal carcinoma.

**Table 2:** Diagnostic confirmation and clinical assessment.

Variable	NPC Group, *n* = 36	Benign Adenoid-Associated Group, *n* = 36
Histopathological assessment performed	36/36 (100%)	36/36 (100%)
Histopathologically confirmed NPC	36/36 (100%)	0/36 (0%)
Histologically confirmed benign adenoid-associated pathology	0/36 (0%)	36/36 (100%)
Flexible nasopharyngeal endoscopy performed	36/36 (100%)	36/36 (100%)

Final group allocation was based on histopathological diagnosis. No *p*-values were calculated for variables used to define the study groups. NPC, nasopharyngeal carcinoma.

**Table 3:** Comparative laboratory and endoscopic characteristics of the study groups.

Variable	NPC Group, *n* = 36	Benign Group	*p*-Value
CRP, mg/L	79.50 (72.50–100.50)	2.08 (1.27–3.36), *n* = 36	<0.001
ESR, mm/h	30.50 (15.75–71.25)	12.00 (8.00–14.00), *n* = 36	<0.001
Leukocyte count, ×10^3^/µL	17.43 (15.06–20.15), *n* = 36	8.50 (8.12–9.38), *n* = 35	<0.001
Platelet count, ×10^3^/µL	262.76 (225.01–301.64)	245.02 (224.27–307.92)	0.545
Loco-regional clinical/endoscopic score	6.50 (5.75–8.00)	2.00 (1.00–3.00)	<0.001
GGT, U/L	32.75 (31.96–42.57)	33.44 (32.59–45.38)	0.171
AST, U/L	24.51 (23.77–24.92)	24.85 (23.80–32.80)	0.287
ALT, U/L	23.20 (22.70–28.92)	25.17 (22.88–25.20)	0.448
Uric acid, mg/dL	4.14 (4.04–4.23)	3.30 (3.21–4.32)	0.006

Between-group comparisons used Mann–Whitney U tests. For leukocyte count, the entry recorded as zero was treated as missing, leaving n = 36 in the NPC group and n = 35 in the benign group. Two additional low values (0.10 and 0.12 × 10³/µL) were retained as recorded but require verification against the original laboratory reports. CRP, C-reactive protein; ESR, erythrocyte sedimentation rate; GGT, gamma-glutamyl transferase; AST, aspartate aminotransferase; ALT, alanine aminotransferase; NPC, nasopharyngeal carcinoma.

**Table 4:** Pooled, within-group, and diagnosis-, age-, and sex-adjusted rank correlations.

Variable pair	Pooled ρ (*p*)	NPC ρ (*p*)	Benign ρ (*p*)	Adjusted Partial ρ (*p*)
CRP–ESR	0.795 (<0.001)	0.777 (<0.001)	−0.087 (0.615)	0.347 (0.003)
CRP–Leukocyte count	0.870 (<0.001)	0.678 (<0.001)	0.255 (0.139)	0.432 (<0.001)
CRP–Loco-regional score	0.814 (<0.001)	0.691 (<0.001)	−0.253 (0.137)	0.226 (0.056)
ESR–Leukocyte count	0.794 (<0.001)	0.928 (<0.001)	−0.091 (0.602)	0.289 (0.015)
ESR–Loco-regional score	0.860 (<0.001)	0.933 (<0.001)	0.132 (0.442)	0.585 (<0.001)
Leukocyte count–Loco-regional score	0.785 (<0.001)	0.950 (<0.001)	−0.837 (<0.001)	0.127 (0.290)

Values are Spearman ρ (*p*-value). Adjusted values are partial rank correlations based on residualized ranks controlling for diagnostic group, age, and sex. Leukocyte pairs used *n* = 71; other pairs used *n* = 72. These exploratory analyses were not adjusted for multiple comparisons.

**Table 6:** Separate age- and sex-adjusted associations with histopathologically confirmed NPC.

Predictor	Scaling Unit	Adjusted OR	95% CI Lower	95% CI Upper	*p*-Value	*n*
CRP	Per 10 mg/L increase	3.45	1.52	7.86	0.003	72
ESR	Per 10 mm/h increase	1.31	0.87	1.98	0.200	72
Leukocyte count	Per 1 × 10^3^/µL increase	4.66	1.37	15.78	0.014	71
Loco-regional clinical/endoscopic score	Per 1-point increase	18.07	1.99	163.91	0.010	72

Separate Firth penalized logistic regression models were fitted for each predictor and adjusted for age and sex. The leukocyte entry recorded as zero was treated as missing and excluded from the leukocyte model, leaving 71 observations (n = 71). OR, odds ratio; CI, confidence interval.

## Data Availability

The de-identified analytic dataset supporting the reported analyses may be available from the corresponding author upon reasonable request and subject to institutional ethics and data-protection approval. Individual source records, original laboratory reports, and potentially identifying clinical data are not publicly available.

## Abbreviations

The following abbreviations are used in this manuscript:

NPC	Nasopharyngeal carcinoma
CRP	C-reactive protein
ESR	Erythrocyte sedimentation rate
GGT	Gamma-glutamyl transferase
AST	Aspartate aminotransferase
ALT	Alanine aminotransferase
ROC	Receiver operating characteristic
AUC	Area under the curve
OR	Odds ratio
CI	Confidence interval
EBV	Epstein–Barr virus
ENT	Ear, nose, and throat
STROBE	Strengthening the Reporting of Observational Studies in Epidemiology

## References

[1] Nath I., Raphael V., Shunyu N.B., Siddiqui A., Chakraborty S., Dey B., Sahoo O.S., Dhar R., Karmakar S. (2026). The triad of nasopharyngeal carcinoma pathogenesis: Host genetics, viral infection, and environmental exposures. Front. Oncol..

[2] Salehiniya H., Mohammadian M., Mohammadian-Hafshejani A., Mahdavifar N. (2018). Nasopharyngeal cancer in the world: Epidemiology, incidence, mortality and risk factors. World Cancer Res. J..

[3] Niedobitek G. (2000). Epstein-Barr virus infection in the pathogenesis of nasopharyngeal carcinoma. Mol. Pathol..

[4] Wang X., Hu D., Li R., Liao W., Li C., Liu Q., Li J., Li Q., Guo W., Wang L. (2026). Epstein-Barr virus-associated tumors: Commonalities in pathogenesis and the tumor immune microenvironment. Front. Immunol..

[5] Low Y.H., Loh C.J.L., Peh D.Y.Y., Chu A.J.M., Han S., Toh H.C. (2023). Pathogenesis and therapeutic implications of EBV-associated epithelial cancers. Front. Oncol..

[6] Purvez A., Mir A., Bashir M., Hytten L., Noori L. (2026). Systemic immunoglobulin changes following adenoidectomy, tonsillectomy, and adenotonsillectomy in patients with chronic tonsillitis and adenoiditis: A prospective study. Cureus.

[7] Szekely D., Zara F., Patrascu R., Dumitru C.S., Novacescu D., Manole A., Mogoanta C.A., Iovanescu D., Iovanescu G. (2025). Histopathological and molecular insights into chronic nasopharyngeal and otic disorders in children: Structural and immune mechanisms underlying disease chronicity. Life.

[8] Kamel R.H., Ishak E.A. (1990). Enlarged adenoid and adenoidectomy in adults: Endoscopic approach and histopathological study. J. Laryngol. Otol..

[9] Huang M., Chen Z., Qin S., Zhou J., Huang Y., Peng S., Huang P., Lin Y., Alenzi M., Huang J. (2024). Gamma-glutamyl transferase to aspartate aminotransferase ratio (GSR) predicts prognoses in patients with colorectal cancer with liver metastasis after microwave ablation. BMC Gastroenterol..

[10] Dhali A., Dahiya D.S., Faisal A.R., Zaman A., Khan F., Biswas J., Bharadwaj H.R., Mandal S. (2026). Persistently elevated gamma-glutamyl transferase and hepatobiliary malignancies: A real-world cohort study. Cancers.

[11] Zhou X., Matskova L., Zheng S., Wang X., Wang Y., Xiao X., Mo Y., Wölke M., Li L., Zheng Q. (2022). Mechanisms of anergic inflammatory response in nasopharyngeal carcinoma cells despite ubiquitous constitutive nf-κb activation. Front. Cell Dev. Biol..

[12] Guo L.-L., Wang H.-Y., Zheng L.-S., Wang M.-D., Cao Y., Li Y., Liu Z.-J., Peng L.-X., Huang B.-J., Shao J.-Y. (2021). Metastasis of nasopharyngeal carcinoma: What we know and do not know. Vis. Cancer Med..

[13] Chen R., Zhou Y., Yuan Y., Zhang Q., He S., Chen Y., Ren Y. (2019). Effect of crp and kinetics of crp in prognosis of nasopharyngeal carcinoma. Front. Oncol..

[14] Tang L., Li X., Wang Y., Tong Y. (2024). Prognostic study of inflammatory markers in nasopharyngeal carcinoma patients receiving intensity-modulated radiotherapy. Cancer Manag. Res..

[15] King A.D., Vlantis A.C., Bhatia K.S., Zee B.C., Woo J.K., Tse G.M., Chan A.T., Ahuja A.T. (2011). Primary nasopharyngeal carcinoma: Diagnostic accuracy of MR imaging versus that of endoscopy and endoscopic biopsy. Radiology.

[16] King A.D., Woo J.K.S., Ai Q.Y., Chan J.S.M., Lam W.K.J., Tse I.O.L., Bhatia K.S., Zee B.C.Y., Hui E.P., Ma B.B.Y. (2019). Complementary roles of MRI and endoscopic examination in the early detection of nasopharyngeal carcinoma. Ann. Oncol..

[17] Wang H., Qi S., Liu C., Qiao Z., Zhang C. (2025). Differentiating early-stage nasopharyngeal carcinoma from adenoidal hypertrophy via SLC40A1 expression and developing a prognostic model for disease progression. Am. J. Cancer Res..

[18] Awawda M., Hanna M., Awawdi A., Salman S., Shirron N., Billan S. (2025). The impact of demographic and clinico-pathological characteristics on recurrence-free survival in nasopharyngeal carcinoma. Cancers.

[19] Szekely D., Zara F., Patrascu R., Dumitru C.S., Barb A.C., Novacescu D., Manole A., Iovanescu D., Iovanescu G. (2026). Inflammatory endotypes of chronic adenoiditis and their impact on persistent middle ear dysfunction: A 2-year retrospective translational study integrating clustering and machine learning approaches. Medicina.

[20] Wang H. (2020). Chronic adenoiditis. J. Int. Med. Res..

[21] Chen L., Alabdullah M., Mahnke K. (2023). Adenosine, bridging chronic inflammation and tumor growth. Front. Immunol..

[22] Peker P., Geçgel A., Özkan O., Gökmen I., Menekse S., Duşgun A., Duman B.B., Çil T. (2025). Systemic immune-inflammation index as a prognostic marker in gastric and gastroesophageal junction cancers receiving perioperative flot therapy. Medicina.

[23] Rizzo A., Vitale E., Maistrello L., Santa K., Santoni M. (2026). Association between systemic immune-inflammatory index (sii) and cancer grading and staging: Evidence from the current scientific literature. Biology.

[24] Lee M.W., Park J.M., Cho I.R., Chung K.H., Kim B.S., Choi J.H., Paik W.H., Ryu J.K., Han K., Lee S.H. (2025). Serum gamma-glutamyl transferase level is associated with the risk of pancreatic cystic neoplasms: A nationwide retrospective cohort study. World J. Gastroenterol..

[25] Venter F.C., Ghitea T.C., Venter A.N., El-Kharoubi A.-F., El-Kharoubi M., Ghitea E.C., Ghitea M.C., Venter A. (2025). The role of dental status in the pathogenesis and severity of peritonsillar and cervical infections. Front. Med..

[26] Chen T., Ma J., Zhou Q., Pang L., Song L., Zuo X., Bai Y., Lian X. (2025). Circulating gamma-glutamyl transpeptidase, systemic inflammation biomarkers and risk of lung cancer in the UK biobank prospective cohort study. Sci. Rep..

[27] Thakur S., Kumar V., Das R., Sharma V., Mehta D.K. (2024). Biomarkers of hepatic toxicity: An overview. Curr. Ther. Res. Clin. Exp..

[28] Fu C., Sun L., Zhang L., Zhou T., Bi Y. (2025). Identification of key biomarkers and potential therapeutic drugs in nasopharyngeal carcinoma based on comprehensive bioinformatics analysis. Discov. Oncol..

[29] Aziz N., Rahmawati L., Cho J.Y. (2025). Identification of diagnostic and prognostic biomarkers in nasopharyngeal carcinoma using integrated transcriptomics and elastic net survival analysis. Open Bioinform. J..

[30] Sobti A., Sakellariou C., Nilsson J.S., Askmyr D., Greiff L., Lindstedt M. (2023). Exploring spatial heterogeneity of immune cells in nasopharyngeal cancer. Cancers.

[31] Fang Y., Wang Y., Yang L., Yuan S., Gu J., Zhou Z., Fang Z., Li Y. (2025). Inflammatory pathways and immune microenvironment in non-small cell lung cancer: Multi-dimensional analysis and machine learning prediction. J. Cell Mol. Med..

[32] Galasso M., Verde L., Barrea L., Savastano S., Colao A., Frühbeck G., Muscogiuri G. (2025). The impact of different nutritional approaches on body composition in people living with obesity. Curr. Obes. Rep..

[33] Gaur Y., Chaudhary A., Abraham A. (2025). Advances in liver tumour diagnosis and treatment: Etiology, classification, and the emerging role of machine learning. IEEE Access.

[34] Shakerdi A.L., Finnegan E., Sheng Y.-Y., Pidgeon G.P. (2025). The multifaceted role of autophagy in nasopharyngeal carcinoma: Translational perspectives on pathogenesis, biomarkers, treatment resistance, and emerging therapies. Cancers.

